# Estimation of Physiologic Pressures: Invasive and Non-Invasive Techniques, AI Models, and Future Perspectives

**DOI:** 10.3390/s23125744

**Published:** 2023-06-20

**Authors:** Sharanya Manga, Neha Muthavarapu, Renisha Redij, Bhavana Baraskar, Avneet Kaur, Sunil Gaddam, Keerthy Gopalakrishnan, Rutuja Shinde, Anjali Rajagopal, Poulami Samaddar, Devanshi N. Damani, Suganti Shivaram, Shuvashis Dey, Dipankar Mitra, Sayan Roy, Kanchan Kulkarni, Shivaram P. Arunachalam

**Affiliations:** 1 Department of Cardiovascular Medicine, Mayo Clinic, Rochester, MN 55905, USA; manga.sharanya@mayo.edu (S.M.);; 2 GIH Artificial Intelligence Laboratory (GAIL), Division of Gastroenterology and Hepatology, Department of Medicine, Mayo Clinic, Rochester, MN 55905, USA; 3 Department of Radiology, Mayo Clinic, Rochester, MN 55905, USA; 4 Microwave Engineering and Imaging Laboratory (MEIL), Division of Gastroenterology and Hepatology, Department of Medicine, Mayo Clinic, Rochester, MN 55905, USA; 5 Department of Medicine, Mayo Clinic, Rochester, MN 55905, USA; 6 Department of Internal Medicine, Texas Tech University Health Science Center, El Paso, TX 79995, USA; 7 Department of Laboratory Medicine and Pathology, Mayo Clinic, Rochester, MN 55905, USA; 8 Department of Electrical and Computer Engineering, North Dakota State University, Fargo, ND 58105, USA; 9 Department of Computer Science, University of Wisconsin-La Crosse, La Crosse, WI 54601, USA; 10 Department of Electrical Engineering and Computer Science, South Dakota Mines, Rapid City, SD 57701, USA; 11 Centre de Recherche Cardio-Thoracique de Bordeaux, University of Bordeaux, INSERM, U1045, 33000 Bordeaux, France; 12 IHU Liryc, Heart Rhythm Disease Institute, Fondation Bordeaux Université, Bordeaux, 33600 Pessac, France

**Keywords:** microwaves, dielectric properties, permittivity, conductivity, microwave imaging, blood pressure, capillary wedge pressure, hepatic portal gradients, intracranial pressures, noninvasive sensors

## Abstract

The measurement of physiologic pressure helps diagnose and prevent associated health complications. From typical conventional methods to more complicated modalities, such as the estimation of intracranial pressures, numerous invasive and noninvasive tools that provide us with insight into daily physiology and aid in understanding pathology are within our grasp. Currently, our standards for estimating vital pressures, including continuous BP measurements, pulmonary capillary wedge pressures, and hepatic portal gradients, involve the use of invasive modalities. As an emerging field in medical technology, artificial intelligence (AI) has been incorporated into analyzing and predicting patterns of physiologic pressures. AI has been used to construct models that have clinical applicability both in hospital settings and at-home settings for ease of use for patients. Studies applying AI to each of these compartmental pressures were searched and shortlisted for thorough assessment and review. There are several AI-based innovations in noninvasive blood pressure estimation based on imaging, auscultation, oscillometry and wearable technology employing biosignals. The purpose of this review is to provide an in-depth assessment of the involved physiologies, prevailing methodologies and emerging technologies incorporating AI in clinical practice for each type of compartmental pressure measurement. We also bring to the forefront AI-based noninvasive estimation techniques for physiologic pressure based on microwave systems that have promising potential for clinical practice.

## 1. Introduction

The pressures in the human body are influenced by muscles, fluids and environmental forces [1]. In surgical and diagnostic procedures, pressure sensors are sensitive tools for assessing and monitoring the physiologic state of an individual. Due to the complexity of the human body, each part has a different physiological markup that undergoes a change in pressure depending on the pathological distinction [1]. Three distinct categories could be created to assess the bodily pressure: low pressure (brain and capillaries), medium pressure (internal organs) and high pressure (joints) [2]. The pressure ranges can be as low as (0–7.5 mmHg) for intracranial pressure to as high as (0–150 mmHg) for left ventricular heart pressure [3]. Currently, we have many quantifiable pressures in the human body, including intraocular, pulmonary arterial, intraluminal, intramuscular, intraurethral and more. In vivo pressure discrepancies in various body compartments play a vital role in physiological regulation. Changes in the normal pressures of the body form the basis for numerous pathologies, for example, the development of acute compartment syndrome following a fracture or soft tissue injury, which causes a block in circulation and eventually leads to necrosis [4]. Such a rise in pressure >30 mmHg is treated as a surgical emergency to prevent loss of the limb [5]. Similarly, in the respiratory system, physiological pressures created by the barriers in the alveoli and pleura are subject to variability due to aging and disease and result in a decrease in muscular strength and elastic recoil [6].

Traditional methods of pressure estimation utilize invasive modalities mostly based on catheterization, as it is the most direct and reliable source of measurement available. These techniques involve the transduction of biological signals into waveforms that form the basis of clinical interpretation. These waveforms are further expanded upon using available technologies to make the interpretation quick and efficient while retaining accuracy. In recent years, innovation in healthcare has allowed us to utilize new technology to perform noninvasive pressure measurements by using various sensor models. Researchers are keen on developing various biomedical sensors based on radiofrequency and microwaves to make diagnosis noninvasive and cost-effective.

Artificial intelligence (AI) has been the new cornerstone of science and engineering in developing machines that can enact human-like thoughts and processes that comprehend large amounts of data. It is the new frontier in medicine and has been incorporated into diagnostic and prognostic models for use in daily clinical practice. It is possible to not only integrate the data into wearable devices but also to predict risk factor models using genetics, behavioral and socioeconomic variables in diagnosing and treating health conditions with the patient’s personalized data [7]. Various AI models have been used for the prediction of disease development, the majority of which are in use for the management of hypertension and cardiovascular risk development.

The purpose of this review is to summarize the physiology of various pressure systems in the body, the existing invasive and noninvasive standards of measurement for each of them and recent developments in using AI for noninvasive measurement and prediction of disease development. Within the existing literature, most work regarding this topic has been solely focused on blood pressure estimation and other compartmental pressures have seldom been discussed. Previous works with similar objectives have discussed the application of artificial intelligence in this field with a limited scope, mainly focusing on one pressure system. This review focuses on advances made in all compartmental pressures for which any AI-based measurement system has been developed, and the studies have been assessed to enumerate any gaps in current research for building similar works in the future. Additionally, future perspectives on the design and implementation of AI-assisted microwave systems for noninvasive pressure estimation are provided for a variety of clinical applications, which is a relatively new topic that is unexplored in the review literature.

## 2. Methodology

The data collection for this literature review was performed by all the authors by using specific search terms on Pubmed, Google Scholar and IEEE Xplore. The inclusion criteria are as follows: (1) Studies discussing/employing artificial intelligence, machine learning and microwave systems, (2) studies discussing/measuring physiological pressures in compliance with measurement standards and (3) studies written in the English language. The subsections dealing with specific physiologic systems in the paper were divided among the authors, who employed terms relevant to the assigned topic combined with “Artficial Intelligence” and “Machine Learning” for their search strategy. Two sets of studies were collected by each author, the first one with those using invasive techniques and the second one with noninvasive techniques.

## 3. Physiology of In Vivo Pressures

This section briefly introduces the mechanisms of various physiological pressures in the body that have clinical relevance and touches on their interpretation and application.

### 3.1. Blood Pressure and Arterial Waveforms

Cardiovascular diseases comprise an extensive group of health conditions and their subsequent complications that stem from pathology in the heart and blood vessels. There is further variation in the rate of disease progression and prognostic response to therapy among genders and various ethnic groups [8]. Hypertension (HTN) has long been cited as the “silent killer” [9]. Between 2017 and 2020, HTN was recorded in 48.1% of adults in the US aged 18 and above, with a documented 33.6 million visits to doctor’s offices and 1.1 million visits to the emergency department [10].

Arterial pressure directly corresponds to cardiac output, arterial elasticity, and peripheral vascular resistance and is measured using an invasive arterial line [11]. The arterial pressure waveform is caused by the outflow of blood from the left ventricle during systole and is followed by diastolic arterial dissipation of the stroke volume [12]. It reflects the change in arterial pressure over time (denoted by dP/dt). Generally speaking, the slope of the waveform is proportional to the dP/dt, which is correlated to the strength of the contractile forces and vice versa [13]. Some studies seem to support the idea that the radial arterial waveform can be predictive of left ventricular function [14,15], although there is contradicting information on the same topic across the literature [16]. In high-compliance vessels, the pressure waveform is characterized by a sharp upstroke, a slow rise to peak, the dicrotic notch, and an exponential diastolic decline [17]. In daily practice, the most commonly used clinical parameters are systolic pressure (SBP), diastolic pressure (DBP) and pulse pressure (PP), which is the difference between the SBP and DBP. It is known to be associated with a higher risk of heart disease and atrial fibrillation [18,19,20]. As compliance decreases down the arterial tree, the pulse pressure is amplified, which is reflected in the waveform as a natural distortion. Since this is not true amplification, peripherally measured PP simply overestimates central PP on the pulse wave analysis [21]. On the other hand, low-compliance vessels such as the peripheral arterioles and capillaries reflect a lower PP, a phenomenon known as damping. 

Pulse wave analysis is also utilized in measuring arterial stiffness and pressures within the cardiovascular system that are of clinical significance, such as the cardiac output. Some of the major applications of it are the monitoring of critically ill patients and the measurement of cardiovascular pressure changes in patients on mechanical ventilation [22,23,24]. Further modalities of pulse wave analysis are discussed in the imaging-based noninvasive blood pressure measurement section of the paper.

### 3.2. Central Venous Pressure

Central venous pressure (CVP) is determined by the combined interaction [25,26] of venous return and cardiac output. In most situations, it is equivalent to the right atrial pressure [27], which eventually fills up the right ventricle and provides preload to the left heart. Normal CVP is below zero when measured in the upright position [28,29]. CVP is an important determinant of the fluid and volume status of cardiac patients and those that are hospitalized in a critical care setting [29,30,31]. Components of the CVP waveform [29] can be used to diagnose various respiratory disorders, cardiac conduction abnormalities, valvular disorders and many other pathologies. One of the most common applications of CVP is during the state of hypovolemia, when the body [32] tries to maintain perfusion by redistributing blood to the vital organs, eventually leading to multiple organ failure. In these cases, fluid administration increases the venous return, which is directly proportional to the CVP. In critical care settings, cardiac function curve monitoring is advised to prevent excessive administration of fluids. Once the curve plateaus, further increasing the fluid does not affect the cardiac output.

### 3.3. Hepatic Portal Pressure

Portal pressure is measured using the hepatic venous pressure gradient (HVPG), the difference between portal vein pressures and the intra-abdominal section of the inferior vena cava [33]. In normal individuals, the HVPG is measured at 2–5 mmHg. A number above 6 mmHg indicates portal HTN, one of the most dreaded sequelae of the cirrhosis of the liver. The clinically relevant state of this complication is diagnosed by a HVPG of >10 mmHg and is also used in assessing the prognosis of the condition [34,35]. Its use can be expanded to predict overall complications of liver disease, including the risk of developing hepatocellular carcinoma [36,37].

### 3.4. Intracranial Pressure

Intracranial pressure (ICP) refers to the internal pressure within the brain consisting of brain tissue, blood and cerebrospinal fluid (CSF). Normal physiologic mean ICP ranges between 7 and 15 mm in adults. The ICP is balanced by the movement of fluids in the brain, such as the inflow of arterial blood, the outflow of venous blood and the production and drainage of cerebrospinal fluid (CSF) [38]. The ICP can increase in cases of intracranial hemorrhage, cerebral edema or the presence of a tumor or mass. Elevated ICP can lead to cerebral ischemia and/or brain herniation, thus, monitoring of intracranial pressure becomes important in certain neurological conditions and traumatic brain injury [39]. The intracranial pressure can be calculated using the cerebral perfusion pressure (CPP) and mean arterial pressure (MAP): CPP = MAP − ICP.

### 3.5. Intrauterine Pressure

A gravid resting uterus consists of a baseline intrauterine pressure created by the elastic recoil of the muscle and fluid within the cavity. During a contraction, the intrauterine pressure changes can give us information on the intensity, frequency and duration of the contraction that can be used during antepartum and intrapartum monitoring of a patient [40].

### 3.6. Intraocular Pressure

Intraocular pressure (IOP) [41,42] is the pressure exerted by the collective volume of aqueous and vitreous humor, choroidal blood vessels, compliance of the sclera and extraocular muscles. Its normal value ranges from 11 to 21 mmHg, with an early morning increase of 2–3 mmHg [43] due to secretions of adrenal cortex hormones. Ocular pressure [44] is altered by various nonmodifiable factors; however, the prime factor is the balance [42] between the production of aqueous humor in the ciliary body and drainage by the canal of Schlemm. According to a case study by Liu et al. [45], a nocturnal elevation of IOP occurs in older individuals, making them more susceptible to conditions of ocular hypertension, such as open angle glaucoma [46]. This occurs due to the change of body position from upright during the day to recumbent at night, thereby increasing the blood flow to the eye [47,48]. Some populations are particularly prone to high ocular pressures, putting them at risk for optic disc damage and further ocular complications, such as glaucoma. These include diabetic individuals, those with elevated glycated hemoglobin and post-menopausal females [49,50,51,52]. Patients that are at high risk of glaucoma can be advised to do mild to moderate exercise [53,54] as an effective preventative therapy to reduce IOP significantly. Some studies [55] also show the relationship between IOP and ICP. The excessive pressure generated during resistance training activities, such as weightlifting, causes an increase in ICP, which causes venous outflow obstruction, thereby increasing the IOP.

### 3.7. Intra-Abdominal Pressure

Intra-abdominal pressure (IAP) is the pressure that is generated by the muscles and organs inside the abdominal cavity. The musculature of the abdomen forms an integral part of various respiratory movements; therefore, the IAP tends to vary with respiration as well as the compliance of the abdominal wall. The normal IAP in adults ranges from 5 to 7 mmHg [56]. Consecutive readings showing a pressure above 12 mmHg can be diagnosed as intra-abdominal hypertension (IIH). This high pressure that occurs mainly in intensive care patients may reduce the blood supply to the major viscera [56] and eventually lead to multiorgan dysfunction [57,58,59]. Although acute coronary syndrome can occur as a complication in any critically ill patient, it is advised [60] not to measure the IAP in these patients unless implicated. Therefore, correct measurement [61] by trained personnel following all the protocols is necessary. As a variable, IAP can independently predict the mortality rate [59,62,63,64] of such patients. 

## 4. Invasive Procedures and AI Applications

The above-mentioned compartmental pressures form the basis of clinical monitoring in the major organ systems of the body. Measurement of these pressures has historically employed invasive techniques due to their precision. This section discusses the existing methods of invasive techniques employed to measure physiological pressures and studies that have used these techniques to build artificial intelligence models that are used for prediction. Most of these methods have several drawbacks, including long and tedious preparation times, local tissue damage due to their invasive nature, and the risk of local or systemic injury and immune reactions, all of which are discussed in the limitations section.

### 4.1. Invasive Techniques

Intensive monitoring of patients requires a continuous estimate of their blood pressure, and the current standard is the use of an invasive arterial line or A-line [65]. During surgeries or ICU monitoring, less than 30% of patients receive this procedure. The risks associated with catheter infections, bleeding and hematoma formation, nerve damage, limb ischemia and pseudoaneurysms require expert clinicians to ensure proper cannulation and avoid erroneous outcomes [66]. Therefore, as a standard of measurement, a noninvasive alternative that could minimize the risks of the procedure would enhance estimation. Cuff and finger-based monitoring systems for this purpose are also widely used, but discrepancies exist due to patient factors such as movement, vasoconstriction, arm sizes and the possibility of measurement only every few minutes [67].

Similarly, right heart catheterization (RHC) is an invasive method to determine the pulmonary and right-sided intracardiac hemodynamic parameters [68,69]. The balloon-tipped pulmonary artery (Swan–Ganz) catheter is used to measure these pressures. During this process, the atrial, ventricular and pulmonary pressures are recorded through an inflated balloon in the form of pulsatile waveforms [69,70,71,72]. RHC is a cardinal diagnostic tool for several cardiac conditions, such as right heart failure [73,74,75,76], tricuspid regurgitation [77], tricuspid stenosis, pulmonary stenosis, and mitral valve diseases [69]. Moreover, it is considered the gold standard for the diagnosis of pulmonary hypertension [78,79]. The procedure for measuring right heart pressures can be combined to also measure HVPG and perform liver biopsies, highlighting the multidisciplinary applications of this method [80,81,82].

The clinical way of diagnosing and monitoring portal HTN is by measuring the HVPG through hepatic vein catheterization via transjugular, transfemoral or transbrachial routes [80]. It can also be used to assess the status of liver deterioration and monitor the response to pharmacologic treatment [81,82,83]. Although measurement of HVPG represents the current gold standard for the diagnosis and monitoring of portal hypertension, it poses a great risk of gastroesophageal varices and the possibility of hemorrhage in the patient [84,85,86].

Catheterization can also be used in gestational females. Intrauterine pressure catheters (IUPC) are used to record and monitor uterine contractions during induction or augmentation of labor with oxytocin and estimate adequate contractions to diagnose the arrest of labor. Uterine contractions can be recorded through other methods such as manual palpation and external tocography as well, but the intrauterine catheter measurement is the only method for gathering quantitative data on the frequency, duration and intensity of the contraction [87]. Similarly, fluid-based systems such as the external ventricular drain (EVD) of the brain are considered the gold standard for ICP monitoring as they deliver accuracy and have the therapeutic advantage of excess CSF drainage. The pressure in the catheter is equivalent to the intraventricular pressure, which is transmitted by a stain-gauge transducer [88]. Another invasive method involves subarachnoid screw insertion with a hole drilled into the skull and the placement of the catheter tip in the subarachnoid space. Implantable micro-transducers are also lesser-known alternatives to measuring ICP [89,90,91,92,93,94,95].

**Figure 1:**
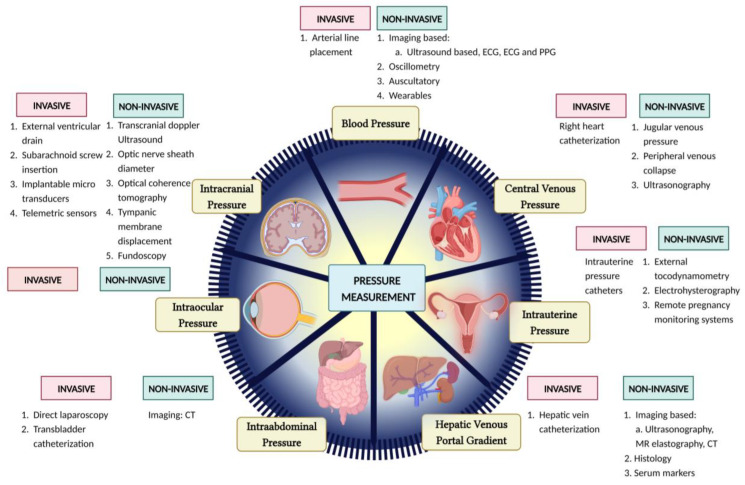
Pictorial representation of several invasive and noninvasive methods for pressure estimation.

IAP is highly important in the clinical world due to the possibility of intra-abdominal HTN, which has a high mortality rate in ICU patients, particularly through abdominal compartment syndrome [96]. Currently, the most accurate method is the highly invasive direct laparoscopic measurement [97], however, it is expensive and requires trained personnel. The most commonly used method in ICU patients is trans-bladder measurement [96]. Figure 1 summarizes broadly the existing models and methods discussed in this paper.

### 4.2. AI Models Using Data from Invasive Techniques

Many studies have shown the use of AI with RHC pressure measurements to be able to predict (1) acute-right ventricular failure in patients with left ventricular assist devices, (2) pulmonary artery wedge pressure using standard chest x-ray [98,99,100,101], (3) mean pulmonary artery pressure in patients with severe tricuspid regurgitation [102] and (4) changes in mean pulmonary artery pressure and pulmonary capillary wedge pressure following vasodilator infusion during RHC showing a seismocardiogram signal recorded by a wearable patch [103,104]. In a retrospective study, Edith Jones et al. [105] presented an interesting methodology to represent the cardiovascular state of both heart failure with preserved ejection fraction and heart failure with reduced ejection fraction patients. The methodology in this study is unique, as it relies on an unsupervised AI model built on features extracted from both RHC and transthoracic echocardiography.

The studies show the potential of AI to combine diverse information such as demographic, clinical (RHC), laboratory, and other data (echocardiography, MRI, etc. that are discussed in later sections) to predict different disease states associated primarily with pulmonary artery pressures. Evaluation of predictions from these machine learning models is not possible without the ground truth defined by RHC. As Conor Hardacre et al. [104] highlight in their review, the limitations and risks of RHC, along with advances in AI, form the motivation for newer and noninvasive diagnostic methods, but there are still circumstances where RHC is valuable, such as the assessment of pulmonary vascular resistance. There have also been AI models constructed for measuring other compartmental pressures, including ICP and IAP [106,107,108].

### 4.3. Limitations of Invasive Techniques

Although catheterization is considered a safe and effective procedure, it also offers complications and limitations. The procedural complications observed are air embolism, arrhythmias, insertion site infections, venous thrombosis, pulmonary pseudoaneurysm, arterio-venous fistula formation and pulmonary infarction. Rarely, perforation of the pulmonary artery (0.03%) is also reported [65,109]. Furthermore, it is required to be performed by highly skilled physicians in order to carry out the whole process without complications and to accurately interpret the waveforms that are generated [110]. These pitfalls make it difficult to access in resource-limited settings.

The limitations of external ventricular drains for ICP measurement include difficult navigation in small ventricles or ventricular compression, the risk of hemorrhage during insertion and the risk of infection with long-term use [88]. Subarachnoid screws increase the risk of local wound infection [39]. While the invasive ICP measurement modalities are the gold standard for accurate measurement, the noninvasive modalities are important for screening for elevated ICP in emergent cases and locations where neurosurgeons are not easily available.

Estimation of the IAP using trans-bladder catheterization can result in local and systemic infection, local pain and residual problems with bladder motion [96]. Similarly, the estimation of intrauterine pressures through catheterization has limited use due to its invasive nature, necessity for rupture of membranes and increased risk of intrauterine infection, uterine perforation, placental disruption, and injury to the fetus [111]. Furthermore, the use of intrauterine pressure measurement is limited to obese patients, where external tocography cannot provide accurate recording [112,113]. It is estimated that 15% of deliveries in the United States use IUPC [114,115].

## 5. Noninvasive and Artificial Intelligence Based Pressure Measurements

To gain an understanding of the topics discussed in this section, it is also essential to briefly define terms often used when constructing AI models. A commonly used subset of AI is machine learning (ML), a type of algorithm that recognizes patterns in input data and uses these patterns to create predictive models. Deep learning (DL) is a subset of ML that predicts patterns and builds on them by performing reanalysis of the results to constantly improve accuracy. Deep learning is often employed in machine learning models to improve the overall performance of AI.

Many studies have found that noninvasive AI-based prediction uses supervised learning algorithms. This method requires a proportion of the data to be entered as labeled input to train the AI model, while the rest of the data is used to test the model. Most studies discussed further use of regression, decision tree, support vector model, K-nearest neighbors, Bayesian model, etc., which fall under supervised learning methods. Unsupervised models, on the other hand, do not include a training set and therefore produce results with lower accuracy and have been seldom applied in this field.

An in-depth analysis of the existing studies that built AI models using noninvasive methods of measurement has been discussed in this section. Since most of the existing body of work in this field deals with estimation and prediction of blood pressure and cardiovascular disease, this topic is discussed in detail with a focus on different modalities of measurement, including imaging-based, oscillation-based, auscultation-based and studies employing wearable technology. This is followed by sections discussing relevant work under other compartmental pressures, and the promising potential of microwave-based technology has been highlighted.

### 5.1. Continuous Blood Pressure Estimation

Application of AI for HTN has been known to predict outcomes in almost all phases of the disease. Various databases are available to utilize for this purpose and construct models for the prediction of the disease. The MIMIC-II and -III Waveform Database available through Physionet includes continuous, high-resolution physiologic waveform records and numeric time series of physiologic measurements [116]. Biobanks linked to electronic medical records are also available across major medical centers (NIH-eMERGE, Mayo Clinic biobank, national UK Biobank) that could be utilized for this purpose [117].

For over a decade, numerous studies have shown that machine learning techniques could predict blood pressure in patients by factoring in their age, weight, body mass index (BMI) and the photoplethysmogram (PPG) signal [118]. In 2013, Samant et al. showed that by using artificial neural networks, their model could diagnose HTN with an accuracy of 92.85% from the data of 981 subjects [117]. Another cohort study by Ye et al. in 2018 showed that using data from electronic health records, such as age, gender, race and underlying disease, could predict the risk of HTN with an accuracy of 91.7% in the retrospective cohort with 823,627 patients and 87% in the prospective cohort with 680,810 subjects [119]. Models conceived so far have been proven to be effective in predicting the incidence of disease in individuals at risk, identifying those suffering from the disease and predicting outcomes that can be correlated with treatment efficacy [120]. One instance is the study performed by Ye, Fu and Hao et al. using statewide electronic health record datasets from the Maine health information exchange network, where it prospectively validated an accurate model that could predict 1-year risk for essential hypertension in these patients [119].

Although HTN is a multifactorial disease, most of the AI models discussed in this study rarely take external factors into account. Most of them utilize raw data available from public databases such as the MIMIC-II and -III systems, where data is available from a list of de-identified patients and therefore only considers the measurements and signals gathered. Some studies have addressed this issue and expanded on it, such as the one performed by Volzke et al. A predictive model using a Bayesian network was constructed from patients with a follow-up period of approximately 5 years, where, in addition to the blood pressure measurements, serum glucose, urine albumin and the rs16998073 genotype were considered for all the patients. The rationale for using this model when compared to other AI models exists in the fact that missing data can be addressed and computed for, which provides it with more potential for clinical applicability [121,122].

#### 5.1.1. Imaging-Based Estimation Techniques

Data from imaging techniques such as ultrasound imaging, transdermal optical imaging, CT imaging and thermal imaging have been applied to construct AI algorithms. B-mode ultrasound imaging linked with various neural networks has been compared to traditional validated methods to ensure the efficacy of AI when applied for the estimation of arterial pressure. 

In 2010, Pessana et al. utilized this technique to measure the instantaneous arterial diameter and, from that, determine the wall artery viscoelastic properties. This was done from data obtained from carotid artery measurements of 10 male patients and it was found that they were no different from the measurement standard used [123]. Similarly, studies performed by Armentano et al. and Graf et al. applied this imaging-based AI to measure carotid wall viscosity through intima media thickness, a measure that is known to increase in thickness due to vascular growth in hypertensives when compared to normotensives [124]. With regression analysis of both studies, high correlation coefficients (r = 0.71 and 0.94) were obtained between the AI-based methods when compared with the traditional metric to determine arterial pressure [125,126].

Expanding on the usage of ultrasonography (USG) images, a combination of imaging results and clinical risk factors (age, sex, BMI, smoking, SBP and DBP) were used by Jamthikar et al. in 2020 to develop a machine learning-based cardiovascular disease (CVD) risk calculator known as “AtheroEdge Composite Risk Score 2.0” (AECRS2.0_ML_). This new tool showed better predictability and superior performance when compared to the FRS [127] and WHO [128] calculators, with an AUROC of 0.87, 0.669 and 0.727, respectively [129].

In addition, a myriad of studies exist that use pulse wave analysis, pulse wave velocity and pulse arrival time for cuffless blood pressure estimation, all of which can be measured using electrocardiography (ECG) and photoplethysmography (PPG) [130,131,132]. Pulse wave velocity (PWV) is the velocity of the pressure wave propagation in the vessels. Kachuee et al. in 2016 utilized data collected using ECG and PPG from the MIMIC database to develop a regression-based AI model to classify blood pressure values. They found that the technology used was able to classify pre-HTN SBP and DBP with an accuracy of 73% and 91%, respectively, and HTN SBP and DBP values with an accuracy of 82% and 98%, respectively [133]. The classification model also proved to be competent both pre- and post-calibration and achieved grade A for the estimation of DBP and grade B for the estimation of MAP according to BHS (British Hypertension Society) criteria [134].

Cano et al. also utilized this data in 2022 to develop a classification system for discriminating between normotensive and hypertensive subjects. Out of the three models developed in the study, the K-nearest neighbors (KNN) model proved most effective, with an accuracy of 93.54%, a sensitivity of 92.31% and a specificity of 94.35% [135]. Another study conducted by Soh et al. also found highest accuracy for the KNN model for the classification of normal and masked HTN, with an accuracy of 97.7% using raw data [136].

The hybrid neural network proposed by Baker et al. also proved to have high efficacy by utilizing the convolutional neural network and long and short term memory model. Raw data in the form of ECG and PPG signals were used as input. Their model achieved low mean absolute errors of 4.41 mmHg for SBP, 2.91 mmHg for DBP and 2.77 mmHg for MAP and also secured grade ‘A’ based on the BHS criteria for blood pressure measuring devices [137].

A relatively promising novel development by Luo et al. in 2019 introduced the use of transdermal optical imaging technology by using hemoglobin signals through a smart phone camera. Data was collected from 1328 subjects and the procedure used a machine learning model to predict SBP, DBP and pulse pressure with 94.8%, 95.8% and 95.8% accuracy, respectively. When demographic features such as age, sex, skin tone, height, weight and race were considered, the model still proved to have an accuracy of >90% for all three variables [138].

In healthcare, the most immediate and helpful use of machine learning is supervised learning, where the data used to train the model are labeled when being inputted and sets of algorithms learn the relationship between input data, such as patient demographics or medical images, and label the outcome data [139]. In some cases, the AI is used to predict the arterial stiffness similar to this study, which used thoracic aorta diameter on nonenhanced CT images in 801 patients and concluded that computer-aided calculation of arterial diameter on chest CT images may be a significant predictor of the risk of cardiovascular disease.

#### 5.1.2. Oscillometry Based Estimation Techniques

Most monitors available for blood pressure estimation are based on oscillometry [140,141,142,143,144,145,146] because of its accuracy and simplicity. Similar to the auscultatory method, this technique also requires a cuff inflation surpassing the threshold systolic pressure, and as it deflates, the oscillometric waveforms (pressure oscillations superimposed on the cuff deflation wave) are recorded [147]. A couple of important issues to understand are that in small studies, the BP estimation is underestimated for SBP and overestimated for DBP [148]; whereas the most popular oscillometric method, the maximum amplitude algorithm, uses data from oscillometric waveforms and empirical coefficients obtained on physiological conditions of arteries and pulse pressures to estimate noninvasive blood pressure estimation (NIBP).

A possible solution that was suggested to overcome the inaccuracies is to apply AI, especially deep learning models as it can help extract all kinds of sounds/waves from each sample and develop a model to establish a relationship between the features that the engineer decides and the reference blood pressure values [147]. Similar to the usual NIBP estimation algorithms, the AI-based ones are divided into two categories: 1. Use the oscillometric waveform data and features from their recordings. 2. Use the oscillometric pulses and extract beat-by-beat features from oscillometric waveforms. The earliest methods were probabilistic methods [145,149,150] where systolic and diastolic ratios were seen as random variables and various classification techniques were used to estimate the blood pressure values. On the other hand, a multiple linear regression method used ten features used in 19 along with area under the oscillometric waveform before and after maximum altitude’s position [151]. Beyond these, an improved technique using deep-belief network-deep neural network [152] to estimate the blood pressure more accurately was proposed, as it can outperform other methods such as maximum amplitude algorithm (MAA), feed-forward neural network (FFNN) and support vector regression (SVR) [153]. It takes advantage of the unsupervised learning step to initialize each layer and uses a backpropagation algorithm to continue the supervised learning steps, which fine-tune the values achieved in the first step [154]. The second classification of AI-based NIBP estimation methods using oscillometric pulses uses classification models such as deep belief networks-deep neural networks (DBN-DNNs) or HMM (hidden Markov model) [155] and long and short term memory recurrent neural networks (LSTM-RNNs) [156].

#### 5.1.3. Auscultation Based Estimation Techniques

One of the oldest methods that has been the gold standard in clinics for BP measurements in NIBP is to use the auscultatory method [157], where a cuff is wrapped around a patient’s upper arm and the observer listens to K-sounds, also known as Korotkoff sounds, using a stethoscope and this principle has been applied to automatic BP systems [158,159]. However, a lot of studies have shown that external noises and personnel differences create differences in the BP values [160,161,162,163]. To reduce that, a convolutional neural network model was used to classify the sounds as K sounds or external noises [159]. The SBP is the cuff pressure at the first pulse of the two consecutive pulses which are classified as K sounds, and the DBP is the moment when no additional K-sounds occur [158]. A study using 277 measurement sets from 42 patients converted each pulse into an image of multiple band-pass filtered signals to increase frequency resolution. Their results were compared with a test set that contained external noises, and they surpassed the baseline model in terms of BP prediction in terms of mean and standard deviation [159]. The AI-based auscultatory NIBP measurements can be classified as either methods that assume Korotkoff sounds are independent and incapable of taking into account the dependencies between consecutive Korotkoff sounds or the methods that extract beat-by-beat features from Auscultatory Waves and can consider dependencies between beats [147].

A vocational technical school has recently used speech recordings consisting of vowels with their corresponding various BP measurements of each participant and used Convolutional Neural Networks-Regression (CNN-R), Support Vector Machines-Regression (SVMs-R) and Multi Linear Regression (MLR) and provided results of 93.65%, 92.15%, 89.43% respectively [164]. A number of machine learning based methods in the existing literature have attempted to some extent overcome the lack of enough training data by data augmentation [152,165]. These use bootstrap technique to augment the training data, while others used elegant deep learning-based methods, including auto-encoders (AEs) and generative adversarial networks (GANs) [147]. Table 1 summarizes some studies carried out in the recent years on various AI technology for the application of predicting and classifying blood pressure using imaging, oscillometry, auscultatory and wearable technology. Some studies [166,167,168,169,170] have used blood pressure as input data to train AI models in predicting subsequent risk of cardiovascular disease development.

#### 5.1.4. Wearables

Wearable technology is at the forefront of healthcare innovation that is widely available to the patients and shows clinical applicability for physicians. Several advancements have been made that reveal the importance of easy to use, readily available physiological monitoring systems that can be used in day-to-day life for health monitoring. Their relative simplicity and universality have allowed for extensive studies to be conducted, such as the model by Zhan et al., that used flexible piezoresistive pressure sensors using a tissue paper model. It succeeded in detecting arterial pressure waveforms, thus giving an accurate measurement of pulse and blood pressure in the patients [171].

Holz et al., designed and constructed the Glabella device in 2017, a wearable pair of spectacles with built in inertial sensors and processors and optical pressure sensors that can record the wearer’s motions and pulse transit time. The angular, superficial temporal and the occipital arteries were used to measure the Pulse Transit Time (PTT) from this device and was shown to predict systolic blood pressure values within ±10 mmHg and for continuous recording of BP. However, it was only a prototype and requires testing on a much larger population to assure its accuracy [172].

**Table 1:** Summary of recent studies on technology applying AI for Blood Pressure prediction and Cardiovascular risk stratification.

Study	AI Model Used	Model Performance Metrics	Limitations
Pessana et al. [123]	Artificial Neural Network (ANN)	Correlated with diameters from B-mode US images; R^2^ = 0.96	Small study population, only male subjects recruited
Jamthikar et al. [129]	ML-based cardiovascular risk calculator-called “AtheroEdge Composite Risk Score 2.0” (AECRS2.0_ML_)	High AUROC (0.87) when compared to FRS (0.67) and WHO (0.72) risk stratification models	No multiethnic cohort used
Kachuee et al. [133]	Regression models (Linear regression, Decision tree, Support Vector Model (SVM), AdaBoost, Random Forest)	High accuracy in prediction of pre-hypertension SBP (73%), DBP (91%) and hypertension SBP (82%), DBP (98%); Grade A in BHS criteria	Parameters collected from database of pre-recorded signals *
Cano et al. [135]	K-Nearest Neighbors (KNN), SVM and Bagging Ensemble Classifier	Distinguish Hypertensive and normotensive subjects; KNN: Accuracy 93.54% SVM: Accuracy 91.35%; Ensemble: Accuracy 90.69%	Parameters collected from database of pre-recorded signals *
Soh et al. [136]	KNN	Classification of normal and masked Hypertension; Accuracy 97.7%	Physical extraction of features, small data size
Baker et al. [137]	Combined Convolutional Neural Network-Long Short Term Memory Network (CNN-LSTM)	Mean average errors (MAE) of 4.41 mmHg for SBP, 2.91 mmHg for DBP, and 2.77 mmHg for MAP; Grade ‘A’ in BHS criteria	Parameters collected from database of pre-recorded signals *
Luo et al. [138]	Unspecified Machine Learning (ML) model	SBP, DBP and pulse pressure with 94.8%, 95.8% and 95.8% accuracy	Cohort only included normotensive individuals, no multiethnic cohort used, no cross-validation done with gold standard techniques
Lee et al. [148]	Bayesian model	Estimated SBP and DBP vs. Auscultatory method, correlation coefficient (r) of 0.86 and 0.87 respectively	Small sample size, manual measurement of BP for reference standard
Lee et al. [149]	Gaussian Mixture Regression	SBP and DBP with MAE of 3.60 and 3.72 respectively	Small sample size, manual measurement of BP for reference standard
Lim et al. [151]	Multiple Linear Regression (MLR) and Support Vector Regression (SVR)	Grade A in BHS criteria for SBP and DBP	Small sample size, only healthy subjects used
Argha et al. [152]	Deep belief network (DBN)-Deep neural network (DNN)	Mean average errors (MAE): MAA (maximum amplitude algorithm) with 9.6 for SBP, 10.8 for DBP; MMSA (Maximum/Minimum slope algorithm) with 9.1 for SBP, 12.9 for DBP.	Parameters collected from database of pre-recorded signals
Chang et al. [159]	CNN	Accuracy of 93.5% for both SBP, DBP	Small study group, faulty recording of microphone sounds, small amplitude sounds go undetected
Chiang et al. [173]	Random forest	Personalised lifestyle recommendations; applying these recommendations showed SBP decreased by 3.8 and DBP decreased by 2.3.	Small study population; high drop off rate.
Ibrahim et al. [166]	AdaBoost	BP prediction tailored to each individual’s vascular properties	Small study population
Wang et al. [167]	Soft Stagewise Regression Network (SSR-Net)	BP prediction	Small study population; only young, healthy subjects used
Lustrek et al. [168]	Multiple algorithms (Decision tree, KNN, Support vector regression, random forest)	Comprehensive self-management of Congestive Heart Failure (CHF), individualized healthcare recommendations	High digital literacy needed for proper application
Huang et al. [169]	Multiple algorithms (Random forest, support vector classifier, naive bayes, generalized linear regression, stochastic gradient descent regressor)	Relative importance of risk factors collected form subjects, tracking prognosis response to treatment	Limited data on high risk group (subjects consisted of 70.2% low risk adults), data from an asian population
Zhou et al. [170]	Random forest classifiers	CVD risk prediction, strong association between wearable derived features and genomic risk markers (regardless of presence of risk factors), strong association between wearable derived features and clinical events.	Limited input on lifestyle factors, short observation period, no gene-environment interaction study conducted
Yoon et al. [174]	ANN	BP estimation compared to real collected ABP values; Grade A on BHS standard	Individual patient factors not considered
Sheng-Kai Ma et al. [175]	Linear regression, Random forest, Support vector regression, Deep Neural Network (DNN), XGBoost	SBP (63.3% accuracy), DBP (80% accuracy)	Small sample size
Sheeraz et al. [176]	Decision Tree	High or Low/Normal SBP or DBP	Small sample size, no external factors considered.

* Limitations: Data collected from ICU patients (older age group, multiple medications in use, only biological signals used, no individual patient data available, no genetic or environmental factors considered).

### 5.2. Other Compartmental Pressures

This section focuses on studies that have explored data from noninvasive methods for pressure measurement of other physiologic pressures and utilization of it to design AI applications.

#### 5.2.1. Hepatic Venous Portal Gradient

A model constructed for HVPG by Marozas et al. relied on selected best-supervised learning algorithms that used a wide set of patient data, including demographic, clinical, laboratory and transient elastography measurements, which were then tested on 21 classification algorithms. Their results showed that the laboratory and transient elastography parameters predicted HVPG ≥ 10 mmHg with 89.72% accuracy with an area under curve (AUC) of 0.96 when compared to the baseline [177].

Convolutional neural network (CNN) is the most popular type of deep learning architecture in medical imaging analysis [178], especially CTs and MRIs. A study using CNNs on CT and MRI images of liver and spleen were established using the training set and internal validation set, respectively, looked at the possibility of diagnosing clinically significant portal HTN. The results showed that the CNN model based on CT and MRI could effectively identify patients with Clinically Significant Portal Hypertension (CSPH) and provide a noninvasive detection method for clinical early screening and diagnosis [179]. Another study using patients with nonalcoholic steatohepatitis (NASH) with compensated cirrhosis hypothesized that HVPG could be extrapolated from liver histology using a convolutional neural network (PathAI, Boston, MA, USA) and accepted its hypothesis as the Machine learning HVPG score was more strongly correlated with HVPG obtained by hepatic vein catheterization than histologically stained biopsies (ρ = 0.47 vs. ρ = 0.28; *p* < 0.001) [180].

#### 5.2.2. Intracranial Pressure

Noninvasive imaging-based techniques use transcranial doppler, optic nerve sheath diameter, optical coherence tomography, MRI, CT and Fundoscopy to measure the ICP. Its drawbacks include inter and intraobserver variability and limited usability in cases where the ultrasound waves cannot penetrate through the skull [181,182,183,184,185,186,187,188,189].

A motion-sensitive MRI can measure pulsatile arterial, venous, and CSF flow. The ratio of pressure to volume change gives the measure of an elastance index that correlates with invasively measured ICP [190,191]. Another study used a CT-determined ratio of CSF volume to total intracranial volume [192,193,194,195,196]. Fundoscopy can be used to visualize the optic disc with a fundoscope, which can identify papilledema due to elevated ICP. A study used fundus photographs to create a grading scale and showed good reproducibility among observers [197]. However, this can only be used as a screening method due to inter-observer variation, and lack of a definite grading scale, and cannot be applied to trauma cases where optic disc swelling is noticeable after some time [198,199].

On the AI forefront for noninvasive ICP measurement, Andrade et al., used a wireless noninvasive monitoring device developed in 2021 that assesses ICP based on skull deformation [200]. It uses a wireless sensor that is placed on the skull, then the transducer picks up signals for sensor pin displacement due to skull deformation seen in elevated ICP, using strain gauge electrical transducer, which is then sent to a digital converter and passes as a Bluetooth signal to the cloud system to be analyzed. The study promises an easy to use, high resolution, and well accessible device that can be used for ICP monitoring. However, further studies are required to validate its accuracy and sensitivity. 

A study by Ye, G et al., applied artificial recurrent neural network using preprocessed ICP data for continuous ICP evaluation on traumatic brain injury patients [201,202]. The accuracy of this model was 94.62% with average sensitivity of 74.91%. This method could be useful for ICU monitoring of patients for elevated ICP. Further randomized controlled trials are necessary to validate its necessity. Another study [203] used a categorical boosting model (CatBoost) to predict a life-threatening event (e.g., mortality) from the onset of intracranial hypertension using ICP and arterial blood pressure measurements in traumatic brain injury patients. This study reasonably predicted mortality in such patients with area under the receiver operating characteristic curve (AUROC) value of 0.7.

#### 5.2.3. Intrauterine Pressure

In gestational women, the superiority of internal tocodynamometry using IUPC over noninvasive external monitoring is not established [204]. External tocodynamometry is commonly used, where an external transducer is fixed to the abdomen with a belt [204]. Drawbacks include reduced sensitivity of the device as compared to gold standard due to maternal obesity, tension on the belt and maternal movement [205]. Alternative methods with improved sensitivity have been developed such Electrohysterography (EHG) [206,207]. This method uses multiple electrodes placed on the patient’s abdomen to record the electricity activity of the uterine muscle that corresponds to the uterine contractions. However, larger randomized controlled trials are required to prove its superiority over the gold standard IUPC [208].

Recent studies have been directed towards tackling the drawbacks of the gold standard and promote remote use via Telehealth. In 2020, Mhajna developed a remote pregnancy monitoring system using a sensor band with a set of biopotential sensors, acoustic sensors, and motion sensors. This device accurately detects fetal and maternal health rates and allows remote monitoring of cardiovascular function. In 2022, the same research group [209] further developed this device, including electrical and acoustic signals in addition to the fetal and maternal heart to accurately detect uterine contractions using a cardiac-derived algorithm. The sensitivity of this device reached 89.8% when compared to the gold standard IUPC. Furthermore, the signals collected from the device are relayed to a cloud storage and signal processing unit wirelessly, and the interpretation can be viewed on a smart cellphone via a mobile app by both the physician and patient, thus allowing for remote monitoring of uterine contractions antepartum and intrapartum. A validation study was conducted for this device by Schwartz et al. [210], that showed the wireless pregnancy monitor (WPM) to have high sensitivity for detecting uterine contractions during labor.

#### 5.2.4. Intra-Abdominal Pressure

Most researchers are trying to use sensors that are attached to body to look at the possibility of monitoring. A study used noninvasive, contactless approach using millimeter waves (30–300 GHz) [211] FMCW (frequency-modulated continuous wave) radar to collect the data and proposed a Pearson-coefficient-guided domain adversarial neural network (PCG-DANN) to process it, which reduces the difference between various individuals and helps to create a mapping relationship between the features and the IAP. Their results using the PCG-DANN out-performed the baselines and other neural network structures, with its mean absolute error lowest among all [212].

### 5.3. AI-Assisted Microwave Systems for Noninvasive Pressure Measurement

Microwaves (MW) are electromagnetic (EM) waves with wavelengths ranging from 1 m to 1 mm and with frequencies ranging from 300 MHz to 300 Ghz. MW is used in various industries, including healthcare. The applications of MW in medicine include disease diagnosis, treatment, drug delivery, hospital waste management, etc. [213]. Researchers are keen on developing various biomedical sensors based on radiofrequency and microwaves to make diagnosis noninvasive and cost-effective. Various noninvasive MW sensors have been developed for blood glucose monitoring based on blood permittivity, reflection, transmission-based techniques, and heart rate monitoring using microwave doppler radar techniques [214,215,216]. However, MW-based technology to measure pressure in the human body is limited.

MW technology has been used widely for pressure measurement in the aerospace industry, mining, and metallurgy [217]. Various MW pressure sensors have been developed, which are helpful in pressure detection in these fields. Recently, researchers have shown interest in EM wave sensors and microstrip patch antennas as promising technology in medicine. It is based on the EM interactions within the human body that helps determine certain physiological features [218]. El Abbasi et al. proposed a wearable, miniaturized microstrip patch antenna to measure blood pressure (BP) using microwave technology. They created a five-layer human tissue arm model and set the frequency range to suit the clinical and engineering standards. They measured BP based on the reflection coefficient over a frequency range and compared it with the standard nonlinear Moens and Korteweg model over varying artery thickness-radius ratios. This study also proposed a novel method to measure pulse transit time to measure BP noninvasively using EM antenna transmission propagation characteristic [218]. Tseng et al. designed a cuffless BP measuring sensor using a microwave near-field self-injection-locked (NFSIL) wrist pulse sensor. The sensor comprised self-oscillating complementary Split Ring Resonator (SO-CSRR) and an amplitude-based demodulator [219]. The pulse transit time is extracted from the pulse waveform’s generated electric field and substituted into the BP computational algorithm to estimate systolic and diastolic BP. This proposed NFSIL BP sensor was compact, had simple system architecture, was cost-effective with high sensitivity, and could potentially help develop cuffless BP sensors for continuous monitoring [219]. 

Farrugia conducted a study to devise a method for dielectric measurements by creating a relationship between the complex permittivity of blood and the concentration of dissolved nitrogen over a wide range of frequencies as a function of pressure [220]. A vector network analyzer (VNA) with an open-ended coaxial probe was used to measure the liquid under test inside an airtight vessel. A significant difference was observed in the relative permittivity of distilled water, with an average increase of around 4% with increased pressure. However, the study has several experimental limitations because of the limited surface area of the pressure vessel [220].

**Figure 2:**
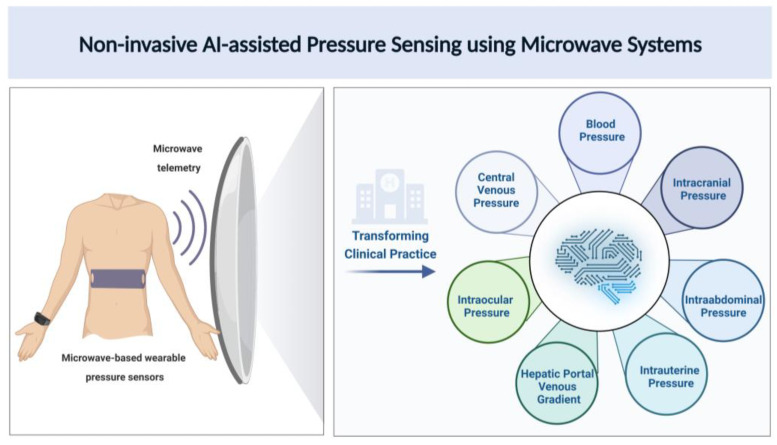
Pictorial representation for noninvasive pressure estimation using AI-assisted microwave systems.

Various studies have shown the relationship between the pulse arterial waveform and BP [221,222]. Research on using EM and radar-based systems to create sensors to measure pulse waveforms to measure BP has been increasing lately. A study used EM waves to measure arterial waveforms and blood volume. EM waves were shown to be sensitive to hematocrit and changes in arterial blood volume [223]. Johnson et al. developed a millimeter-wave radar-based wearable device for measuring arterial pulse waveforms at the wrist. They measured five healthy subjects and showed that pulse waveforms with distinct characteristics of arterial pulses and heart rate matched with ECG measurements. With future developments and improvements, this can potentially be used to monitor blood pressure [224]. Another study by Lee et al. used continuous Doppler microwaves to measure arterial wall movements and pulse pressure to develop a microprocessor-based noninvasive arterial pulse wave analyzer. The results showed that the analyzer could accurately detect arterial wall movement and the pulse wave contour [225]. These studies show that microwaves can be used to measure pressure and can be used to develop sensors for noninvasive continuous monitoring, which is a critical clinical need. Further research is needed in the appropriate development of microwave technology for promising and more accurate pressure sensors to improve healthcare. Figure 2 shows an illustration of novel AI-assisted microwave systems in terms of microwave sensors, microwave telemetry for data transmission and microwave AI-data analytics for noninvasive pressure sensing towards making a clinical impact to improve patient practice.

As shown in Figure 2, novel microwave-based wearable blood pressure sensor designs can provide an easy and reliable way for noninvasive pressure measurement for a variety of clinical applications. Technology innovations in novel microwave antennas leveraging AI-based metamaterial designs and frequently selected surfaces are warranted for optimizing blood pressure sensing. Microwave telemetry systems can enable large-scale wireless data transmission to seamlessly integrate with clinical workflows. Large amounts of big data obtained from continuous blood pressure monitoring can be processed with AI-assisted tools for noninvasive, real-time assessment of physiologic pressures.

## 6. Discussion

As an emerging field in medical technology, artificial intelligence has been incorporated into analyzing and predicting patterns of body pressure that have clinical applicability—both in hospital settings for healthcare providers and at-home settings for ease of use for patients. An analysis of the studies discussed in this paper proves that supervised artificial intelligence models are preferred in this field due to simpler computational methods and higher accuracy of the obtained results. Utilizing this technique, most models discussed in this paper have achieved significant accuracies and show strong potential.

The relatively exciting applications of AI for blood pressure estimation using speech recordings and vowel sounds [164] and transdermal optical imaging [138] show promising potential for day-to-day use. Photoplethysmogram and ECG signals are currently the most reliable and widely researched inputs for the development of BP prediction models due to the open-access databases available on the web [116]. The application of AI has made cardiovascular health accessible to everyone in the form of wearables, which provide data from heart rate, sleep cycles, and PPG signals that can be transduced to predict BP. Continuous estimation of cardiovascular pressures is the next big challenge in this field and has already seen numerous developments. Although widely researched, the multifactorial nature of hypertension and cardiovascular disease makes them tough ailments to predict for AI. Due to the interplay of environmental and genetic factors, a complete study considering all potential influencers has yet to be conducted, including subject age, height, weight, BMI, sex, race, genetic predisposition, family history, social history, environmental factors, sleeping habits, stress levels, pre-existing health conditions and medication use. Despite the myriad of studies in existence that utilize information from databases, it should be realized that the results from these studies cannot be applied in practice due to the lack of external validity. Most of the data utilized are from unidentified subjects that are already hospitalized and have been on steady pressure control medication for decades. Further, factors that are easily overlooked and seldom addressed in most trials, including racial nonheterogeneity, disproportionate inclusion of diseased and nondiseased subjects, the lack of diversity in skin color among subjects for transdermal imaging, the use of faulty technology and the lack of homogenous comparison models, should be realized and incorporated in the future.

The more complicated compartmental pressures, such as the hepatic venous portal gradient and central venous pressures, that require catheter implantation have yet to be initiated in the field of noninvasive applications. Transabdominal biopsy and transjugular catheterization remain the most ubiquitously used parameters for HVPG measurements. However, ultrasonography techniques such as transient and shear wave elastography, MR elastography and abdominal CT have seen an increase in the frequency of use for screening and prediction, although invasive methods remain the gold standard. Recent AI models for clinically significant portal hypertension use CT imaging-based auto machine-learning and compare the results to standard imaging and serum-based tools, with the proposed method outperforming the latter [226]. Similarly, although AI models exist for prediction of central venous pressures that utilize RHC data [227], the proposed noninvasive models have not been shown to outperform the gold standard of invasive monitoring. An ANN model constructed by Moinadini et al. in 2019 used several clinical parameters, including the heart rate, SBP, caval index, lactate clearance and shock index, all of which showed an individual positive correlation with invasive CVP monitoring measurements. This model showed a sensitivity of 100% for normal CVP and 91% for elevated CVP (>8 mmHg) [228].

In the same domain, monitoring of intracranial and uterine pressures is slowly moving towards artificial intelligence methods by incorporating biosensors and wireless connectivity that allows for remote, noninvasive testing. Further randomized controlled trials are necessary to validate its superiority over the gold standard and establish newer protocols using modern technology.

## 7. Conclusions

Preliminary studies show promising results in the usefulness of AI for noninvasive pressure measurement, though more research is warranted. Currently, most scholarly work in this field exists for the measurement of blood pressure and arterial waveforms. It has also been proven that it can be applied to smaller compartmental pressures, such as IAP, IOP, IUP and ICP, but research on that front remains limited. To date, minimal research has been reported on clinically applied artificial intelligence models for noninvasive intra-abdominal and intraocular pressures.

The limitations of most study models elucidated the need for the inclusion of extended data to obtain greater accuracy in future models and employ them for clinical translation. Further improvement in noninvasive pressure measurement methods is necessary, with the accuracy of readings and patient safety as the primary goals. Additionally, microwave imaging-based noninvasive pressure measurement has a lot of untapped potential in this field, as elucidated by the studies discussed prior to this review. Therefore, technological innovations are required for microwave antenna designs and AI applications of microwave data for noninvasive pressure estimations. In conclusion, the AI frontier shows huge potential for better access to healthcare for patients and promises simple, safe and accurate readings for application in pressure measurement.

## Data Availability

The review was based on publicly available academic literature databases.
